# Level of diagnostic agreement in musculoskeletal shoulder diagnosis between remote and face‐to‐face consultations: A retrospective service evaluation

**DOI:** 10.1002/hsr2.2060

**Published:** 2024-04-21

**Authors:** Louise Cockburn, Gill Baer, Jenna Rhodes

**Affiliations:** ^1^ Division of Dietetics Nutrition Biological Sciences Physiotherapy Podiatry and Radiography Queen Margaret University Musselburgh UK

**Keywords:** diagnosis, musculoskeletal disorders, physiotherapy, remote, shoulder, telemedicine

## Abstract

**Background and Aims:**

To determine the level of diagnostic agreement between remote and face‐to‐face consultation in assessing shoulder complaints.

**Methods:**

A retrospective service evaluation with three groups of patient data; those assessed only face‐to‐face (group 1), remotely then face‐to‐face (group 2), remotely only (group 3). Patient data were extracted from 6 secondary care shoulder Advanced Physiotherapy Practitioner's (APPs) records, covering six sites. Three‐hundred‐and‐fifty‐nine sets of patient data were included in the final evaluation. The main outcome measure was the percentage of agreement between diagnosis at initial and follow‐up consultation, when assessed by APPs across the three groups. A Pearson *χ* ^2^ test was used to assess the relationship between the method of consultation and the level of diagnostic agreement. Diagnoses were categorized as either the same, similar, or different by an independent APP. Secondary outcome measures investigated whether age or the length of time between appointments had any effect in determining the level of diagnostic concordance.

**Results:**

There was exact agreement of 77.05% and 85.52% for groups 1 and 3, respectively, compared with 34.93% for patient data in group 2. Similar clinical impressions across both initial and follow‐up were seen 16.39% of the time in group 1, 7.24% of the time in group 3, and 36.99% in group 2. Lastly, the percentage of times a diagnosis was changed between initial and review appointments occurred in only 6.56% of group 1 contacts, 7.24% of group 3 contacts, but 28.08% of the time in group 2.

**Conclusion:**

There was a large mismatch in the diagnosis of musculoskeletal shoulder complaints, when patients are initially assessed remotely and then followed‐up in‐person. This has implications for the future provision of shoulder assessment in physiotherapy.

## 1 INTRODUCTION

Musculoskeletal physiotherapy has seen a dramatic surge in the use of remote consultations since the onset of the coronavirus pandemic. This has been done to combat waiting lists whilst keeping both patients and clinicians safe.^1^, ^2^, ^3^, ^4^, ^5^ Remote consultations fall under “telehealth,” and are defined as over the phone, via video link or online.^6^ The NHS recovery plan drives an onward trajectory of incorporating digital communication between healthcare staff and patients.^7^

Many studies have examined remote consultations in healthcare, mostly exploring patient adherence to health interventions, overall satisfaction and acceptance of clinicians and service users, health economics, and the time burden appointments place on patients.^2^, ^8^, ^9^, ^10^, ^11^, ^12^ Findings have mainly been positive, indicating a place for telehealth in the future provision of day‐to‐day healthcare.

Within current telehealth research, however, there is a scarcity of literature exploring the level of diagnostic agreement between face‐to‐face and remote consultations when assessing musculoskeletal conditions. Face‐to‐face consultations have been described as the “gold standard” in determining an accurate musculoskeletal diagnosis.^13^ This may be due to both the complexity of pain^14^ and the growing body of research indicating incidental findings of limited clinical significance on imaging.^15^, ^16^, ^17^, ^18^ Moreover, there are few studies considering the shoulder complex which has been described as a “diagnostic challenge”^19^ and “difficult” to analyze.^20^ Of the existing research examining diagnostic agreement between remote and in‐person consultations, the shoulder complex has seen less favorable results when compared to other areas of the body,^4^ with 78.6% agreement of a same or similar diagnosis for the shoulder, compared to 92.9% for the lumbar spine.^21^

Studies demonstrating high levels of agreement between remote and face‐to‐face assessments often contain numerous methodological weaknesses including a lack of blinding of all involved, small sample sizes, a lack of acknowledgment to barriers or facilitators^4^ and research undertaken in laboratory rather than clinical settings.^21^, ^22^ A recent systematic review recommended future research should generate data from real clinical populations in target environments.^13^

The acceptability of telehealth as equal to face‐to‐face consultations is contentious.^23^, ^24^, ^25^ A lack of experience and knowledge of telehealth systems, along with inadequate technology infrastructure in healthcare facilities, have been suggested as potential barriers.^8^, ^26^, ^27^, ^28^, ^29^ A cross‐sectional qualitative survey of allied health clinicians working through the covid‐19 pandemic, revealed multiple complaints regarding the inability to “properly” assess patients.^1^ Reported limitations included the loss of palpation skills, poor camera angles creating difficulty observing patients, and trouble conducting special tests. Alternatively, Tanaka et al.^5^ concluded that telehealth assessment of shoulder function and range of motion can be done “adequately.” A review examining special orthopedic tests found disparity in the percentage agreement between remote and face‐to‐face orthopedic tests for the shoulder and ankle at 76% and 99.3% respectively.^13^ Similarly low levels of agreement between telehealth and face‐to‐face assessment for neurodynamic testing of the shoulder (56.1%) and fair to moderate levels of agreement for pain, swelling, and scarring were found.^13^ These findings are endorsed by Turolla et al.,^4^ who query accurate diagnosis and ability to exclude red flags in the absence of palpation or performing specific tests.

Overall, there is currently a lack of robust evidence to conclude that the shoulder complex can be assessed remotely as safely and accurately, as can be done face‐to‐face. The change in musculoskeletal physiotherapy delivery since the start of the pandemic has resulted in a greater reliance on remote consultations. The aim of this retrospective service evaluation was to assess the level of diagnostic agreement between remote and face‐to‐face shoulder assessment. By comparing the levels of agreement between remote‐only appointments and face‐to‐face‐only appointments, any differences seen between those groups and face‐to‐face followed by remote appointments might be inferred as being due to the method of consultation.

## 2 METHODS

### 2.1 Design

This was a retrospective service evaluation investigating the level of agreement between remote and face‐to‐face consultation for the diagnosis of conditions affecting the shoulder. The people undertaking the assessment were Advanced Physiotherapy Practitioners (APPs) in the Integrated Musculoskeletal (imsk) shoulder and elbow team. APPs are highly skilled clinicians with a wealth of experience and elevated knowledge and critical thinking skills.^30^ APPs who specialize in the shoulder would therefore be considered the clinicians best able to demonstrate highest levels of agreement between in‐person and remote shoulder assessment.

### 2.2 Data

This evaluation used data from patients initially assessed within the imsk service between January 1, 2020 to December 31, 2020 and the subsequent follow‐up consultation data. The data was extracted from routinely kept excel spreadsheets, containing information related to patients presenting to the APPs with shoulder complaints only (e.g., not referred from another source) and electronic patient records. Routinely collected data such as patient's age, sex, diagnosis, and assessment date, along with diagnosis were analyzed.

**Figure 1:**
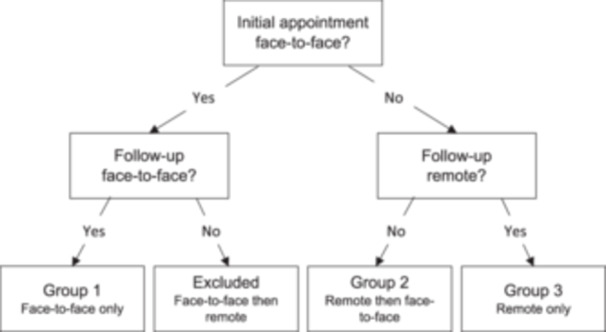
Flowchart of patient data categorization.

From patient records, people who had been both assessed and followed‐up face‐to‐face by the imsk team were identified as group 1. Data from people initially assessed remotely (by telephone or videoconferencing equipment) and who then went on to have a face‐to‐face review, were identified as group 2. Group 3 data consisted of people where both first and second appointments with the APP were remote (Figure 1).

The days between remote and face‐to‐face consultation were also recorded as some conditions may masquerade as another until further symptoms develop.^31^ Therefore, it was useful to examine if longer lapses between appointments led to a higher mismatch of diagnosis.

The patients' sex and age were included in the evaluation to evaluate if the data were normally distributed between groups and to observe if there were any trends for improved consensus in the clinical impressions with a certain age group, particularly when using remote methods to assess and review patients. Age groups were defined as under 45 years, 45–64 years, and over 65 years at the time of initial assessment, due to UK computer literacy rates.^32^

Data had to meet the following conditions for inclusion:

- Data from persons aged ≥18 years old.
- Presenting/referred with unilateral or bilateral shoulder symptoms.

Data were excluded for the following reasons:

Notes included a diagnosis of any cognitive or communication issues that would interfere with assessments (e.g., dementia, deaf).

- No objective assessment.
- Any patients assessed initially by one clinician and followed‐up by another, as this may have introduced confounding variables, for example, interrater reliability.
- Duplicate data sets across the excel spreadsheets.
- Patient only attended one appointment.

### 2.3 Procedure/process

The APP's clinical impression documented in the patient's electronic patient record or indeed, the absence of a diagnosis if the notes offered none, were recorded for both the initial and follow‐up consultation. These were then analyzed by an independent, blinded APP, not working in the imsk shoulder and elbow team. The independent, blinded APP rated the clinical impression between the initial and follow‐up consultation as either the same, similar, or different, on the following basis:

- Same = clinical impressions are an exact match at initial and follow‐up consultations.
- Similar = same structure/condition but minor omissions/additions to either initial or follow‐up clinical impression.
- Different = different structure or condition recorded at initial and follow‐up consultation.

In the absence of a documented clinical impression at the follow‐up appointment, it was assumed that the clinician had not changed their diagnosis and therefore was considered as the same, across all three groups.

After reviewing the independent, blinded APP's categorization, the author asked for clarification on nine sets of data. Any disagreements in the categorizations of data were resolved by an orthopedic consultant physiotherapist, who specialized in the management of shoulder conditions.

### 2.4 Analysis

To determine the viability of using remote shoulder assessment as a suitable alternative to face‐to‐face consultation, diagnostic concordance data between appointments were appropriate for both descriptive and interferential statistical analysis. For the latter, the two‐tailed *χ* ^2^ test of independence was selected as the data were nominal.^33^ These results, along with descriptive statistics to summarize the characteristics of patient data, were generated using IBM SPSS Statistics Version 23 program (IBM) and Microsoft Excel for Microsoft 365 MSO, Version 2112.

## 3 RESULTS

To assess the level of diagnostic agreement between remote and face‐to‐face consultation in assessing shoulder complaints in this retrospective evaluation, data sets were collected and analyzed as described below.

### 3.1 Patient data

**Figure 2:**
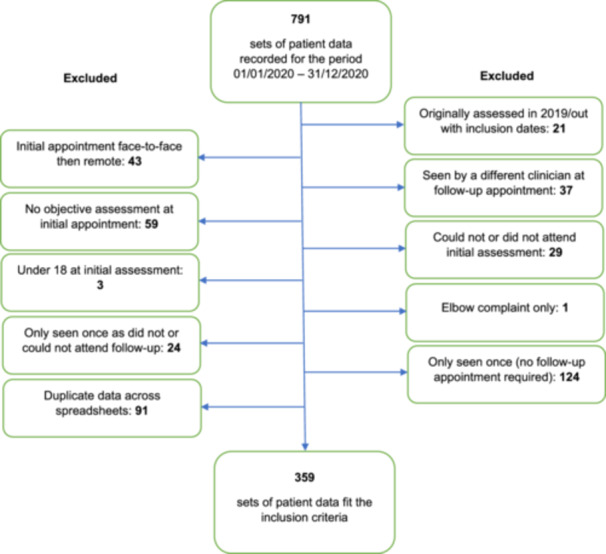
Flowchart depicting inclusion and exclusion of patient data.

Figure 2 presents a flowchart, depicting the patient data included and excluded in this evaluation. Originally, there were 791 sets of patient data recorded for the period January 1, 2020 to December 31, 2020. In total, 432 sets of patient data were excluded, leaving 359 sets of patient data to be included for analysis.

### 3.2 Characteristics of patient data

Table 1 depicts the characteristics of the included patient data sets. Overall, the mean age was 54.02 years (SD: 12.38), with the youngest patient data included 21 years old and the oldest 83 years old. The majority of patient data included was derived from females (59.05%). Histograms, Q–Q plots, and boxplots showed data to be normally distributed (Appendix A).

**Table 1:** Characteristics of patient data.

Characteristics	Face‐to‐face only (group 1), *n* = 61	Remote then face‐to‐face (group 2), *n* = 146	Remote only (group 3), *n* = 152	Total *n* = 359
Age in years, mean (SD)	55.82 (12.4)	54.79 (12.85)	52.56 (11.81)	54.02 (12.38)
Sex				
Female, *n* (%)	35 (57.34)	86 (58.9)	91 (59.87)	212 (59.05)
Male, *n* (%)	26 (42.62)	60 (41.1)	61 (40.13)	147 (40.95)
Days between appointments, mean (SD)	46.39 (68.12)	34.51 (48.15)	37.99 (32.21)	38 (46.6)

### 3.3 Primary outcome: Level of agreement between remote and face‐to‐face methods of assessment

The same clinical impressions were documented in 77.05% and 85.52% for groups 1 and 3, respectively. This contrasts with 34.93% for group 2, patients who were initially assessed remotely then followed‐up in‐person.

Patients notes that documented a similar clinical impression across both initial and follow‐up were seen 16.39% of the time in group 1, 36.99% in group 2, and 7.24% of the time in group 3.

**Figure 3:**
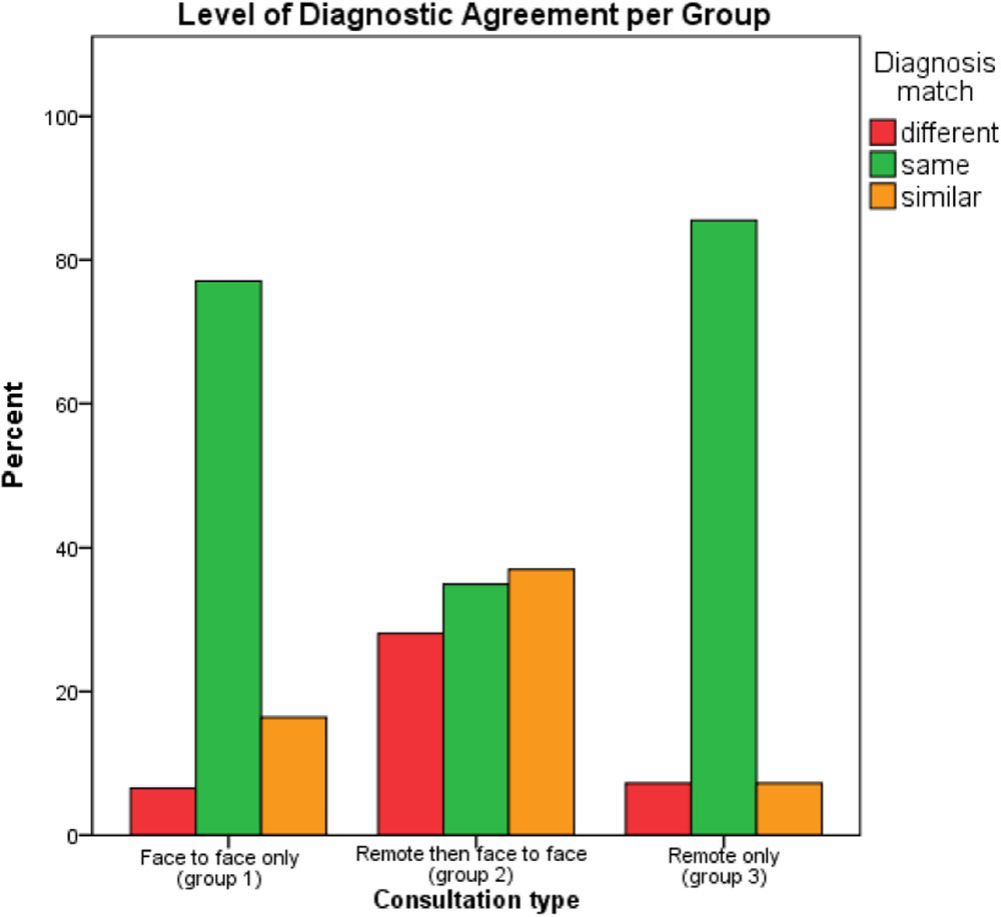
Level of diagnostic agreement between each group.

Lastly, the percentage of times a diagnosis was changed between initial and review appointments occurred in only 6.56% in group 1, 7.24% in group 3, but 28.08% of the time in group 2. These results can be seen in a clustered bar chart (Figure 3).

A *χ* ^2^ test of independence was calculated comparing the frequency of each of the levels of diagnostic agreement with the three consultation types (Tables 2 and 3). The Pearson *χ* ^2^ value found a significant interaction (*χ* ^2^_[4]_ = 88.99, *p* < 0.00001),^34^ demonstrating a less than 0.001% probability that the results above occurred by chance. This suggests that the method of consultation and level of diagnostic agreement are related.

**Table 2:** Crosstabulation for the level of diagnostic agreement and method of consultations.

		Diagnosis match		
Group		Same	Similar	Different
1	Actual count	47	10	4
	Expected count	38.7	12.7	9.5
2	Actual count	51	54	41
	Expected count	92.7	30.5	22.8
3	Actual count	130	11	11
	Expected count	96.5	31.8	23.7

**Table 3:** *χ* ^2 ^test for independence and symmetric measures.

	Value	Degrees of freedom	Asymptotic significance (two‐sided)
Person *χ* ^2^	88.992	4	0
Likelihood ratio	92.474	4	0
Number of valid cases	359		

Symmetric measures		Value	Approximate significance
Nominal by nominal	Phi	0.498	0
	Cramer's *V*	0.352	0

### 3.4 Secondary outcomes: Difference in diagnostic agreement across different assessment methods, dependent on age

When separating the results by age into groups of people <45 years old, 45–64 years old, and >65 years old, there was a higher rate of different diagnoses being recorded between sessions in the older age group, at 25%. This is compared to a different diagnosis in 13.78% of the 45–64‐year‐old patient data and 12.86% for the youngest cohort.

Group 2 resulted in the largest discordance in clinical impressions between appointments with 10% in the <45s, 10.22% of the 45–64‐year‐olds, and 17.19% of the >65s.

### 3.5 Secondary outcomes: Difference in diagnostic agreement depending on length of time between appointments

The longest delay between appointments was 344 days in group 1, 352 days in group 2, and 175 days in group 3 (Table 4). Of these, both initial and second clinical impressions were either the same or similar, appearing to demonstrate that the length of time between appointments did not significantly alter the APPs clinical impression.

**Table 4:** Longest number of days between appointments, for each level of diagnostic agreement.

Level of diagnostic Agreement	Longest time between appointments (days)		
	Face‐to‐face only (group 1)	Remote then face‐to‐face (group 2)	Remote only (group 3)
Same	267	332	175
Similar	344	352	28
Different	35	111	83

Table 5 presents descriptive statistics for the data sets that indicated there was a different clinical impression between first and follow‐up appointment. It demonstrates no major differences in the mean or median days between each of the three groups. This reinforces any differences seen between the groups, may be due to the method of assessment, rather than the time elapsed between appointments.

**Table 5:** Descriptive statistics for different clinical impression recorded at follow‐up appointment.

Days between appointments	Consultation type		
	Face‐to‐face only (group 1)	Remote then face‐to‐face (group 2)	Remote only (group 3)
Mean	24	25.41	25.18
Lowest value	7	0	1
Highest value	35	111	83
Median	27	22	27

The primary aim of this evaluation was to determine the level of agreement between remote and face‐to‐face consultations when examining the shoulder complex. Descriptive statistics demonstrated a difference between diagnosis in group 2, with a different clinical impression documented at initial and follow‐up appointments 28.08% of the time. This contrasts with the face‐to‐face‐only and remote‐only groups, with different clinical impressions documented 6.56% and 7.24%, respectively. A *χ* ^2^ test of independence suggested with a high level of probability that the method of consultation and level of diagnostic agreement are related.

## 4 DISCUSSION

The purpose of this retrospective service evaluation was to assess the level of agreement between remote and in‐person consultations in assessing the shoulder complex. The findings of this evaluation have uncovered some notable results. When pooled together, the diagnoses recorded as the same or similar between initial and return appointments for groups 1 and 3, come to 93.44% and 92.76%, respectively. In stark comparison, this is only 71.92% for group 2. This demonstrates a different diagnosis in 28.08% of cases between the initial remote consultation and the subsequent in‐person follow‐up, indicating over one in every four clinical impressions had some uncertainty. It can be argued that this might indicate a considerable deficit for accuracy of remote consultation when analyzing shoulder complaints, even by APPs, and could lead to the mismanagement of patients with shoulder problems. Future research is advised to assess the variability in diagnostic agreement with less experienced physiotherapists. Furthermore, the rate of different diagnoses recorded between sessions was higher in the over 65‐year‐old age group, compared with younger cohorts, irrespective the method of consultation. This may be due to the likelihood of increasing co‐morbidities^35^ and therefore complexity in making a correct diagnosis.^36^ It could also be argued that it may be due, in part, to a lower computer literacy rate in this age group.^32^

The result of patients not receiving a correct diagnosis and therefore being mismanaged may potentially lead to patients experiencing pain, disability, and a lack of social participation including employment for longer than necessary. There is evidence to suggest the longer a person is absent from their work, through illness or injury, the less likely they are to return to the workplace.^37^, ^38^ This obviously has serious financial and quality‐of‐life implications for those affected, stretching far beyond the clinician's treatment room. In addition, therapists' confidence and overall job satisfaction may also be negatively impacted upon. One clinician from a qualitative survey advised that a lack of confidence in their diagnosis affected their treatment planning.^1^ This is echoed by Schutz et al.^39^ that remote consultations were considered “not fit for purpose” when attempting to make a diagnosis in primary care. The result of mismanaged patients could be a delay in appropriate treatment, poorer patient outcomes^40^ and ultimately, higher medicolegal costs for health boards.^41^ The literature supports careful consideration be given to more complex patient cases, to have them initially assessed face‐to‐face^4^ and for a balance to be struck between remote and face‐to‐face consultations to mitigate against the aforementioned risks.^42^ Given the results of this evaluation, these authors would go further to suggest, where possible, all initial consultations for shoulder examination are carried out in‐person initially, particularly with the older population.

## 5 LIMITATIONS

Initially this study was planned to be prospective, however due to the Covid‐19 pandemic, it was to prove problematic to obtain ethical approval for a prospective study where further controls could have been put in place. A prospective study may have resulted in less missing data in relation to recording a clinical impression at both initial and follow‐up appointments. An assumption was made in the absence of a clinical impression at follow‐up, that it was essentially unchanged, however there is no certainty that this was the case. The only way of mitigating this was that this assumption was upheld across all three groups.

There were multiple instances whereby the APP did not carry out an objective assessment when assessing the patient remotely, instead choosing to make the following appointment face‐to‐face to reach a diagnosis. This meant a raft of data could not be included. One could cautiously argue that in these instances, the clinician felt they would garner more from a face‐to‐face assessment than they would remotely. Indeed, there were numerous comments in APP's notes expressing their uncertainty when carrying out remote consultations, even in the cases where initial and follow‐up consultations clinical impressions were the same. This is supported by Rosen et al.^42^ whose qualitative study describes inaccurate, misunderstood, or missing information between GPs and patients during remote consultation. Rosen et al.^42^ further revealed that clinicians reported stress due to the uncertainties surrounding remote appointments and that remote consultations risk inadequate assessment and therefore an incorrect diagnosis.

## 6 CONCLUSION

This retrospective service evaluation has demonstrated there was a considerable difference in the diagnosis of musculoskeletal shoulder complaints when patients are initially assessed remotely, and then followed‐up in‐person by specialist shoulder and elbow APPs. It is the recommendation of these authors that where possible, initial appointments should be conducted face‐to‐face, to have the greatest opportunity at reaching a sound diagnosis. Further prospective research into the levels of agreement between remote and in‐person shoulder assessment with physiotherapists of varying experience would be beneficial and have potential implications for the future provision of shoulder assessment in physiotherapy.

## AUTHOR CONTRIBUTIONS

**Louise Cockburn**: Conceptualization; data curation; formal analysis; investigation; methodology; writing—original draft; writing—review and editing. **Gill Baer**: Resources; supervision; writing—review and editing. **Jenna Rhodes**: Resources; supervision; writing—review and editing. all authors have read and approved the final version of the manuscript.

## CONFLICT OF INTEREST STATEMENT

The authors declare no conflict of interest.

## ETHICS STATEMENT

Ethical approval to conduct this evaluation was obtained from Queen Margaret University, Edinburgh on 19 October 2021. Approval from NHS Lothian Caldicott Guardian (CG/DF/2190) and Edinburgh Health and Social Care Partnership's Quality Improvement team was granted before this. Ethical approval was granted on the provision that raw data would be stored confidentially and kept for a 5‐year period on Queen Margaret University, Edinburgh's servers.

## TRANSPARENCY STATEMENT

The lead author, Louise Cockburn, affirms that this manuscript is an honest, accurate, and transparent account of the study being reported; that no important aspects of the study have been omitted.

## Data Availability

The lead author, Louise Cockburn, had full access to all data in this study and takes complete responsibility for the integrity of the data and the accuracy of the data analysis.

## APPENDIX A NORMALITY OF AGE DATA ACROSS ALL THREE GROUPS IN Q–Q PLOTS AND BOXPLOT

Normal Q–Q plots

**Boxplot**
